# Associations of genetic variants of endothelin with cardiovascular complications in patients with renal failure

**DOI:** 10.1186/s12882-017-0707-2

**Published:** 2017-09-07

**Authors:** Chih-Chin Kao, Shih-Ying Cheng, Mei-Yi Wu, Shu-Chen Chien, Hsing-Fang Lu, Yu-Wen Hsu, Yan-Feng Zhang, Mai-Szu Wu, Wei-Chiao Chang

**Affiliations:** 1 Division of Nephrology, Department of Internal Medicine, Taipei Medical University Hospital, Taipei, Taiwan; 2 Graduate Institute of Clinical Medicine, College of Medicine, Taipei Medical University, Taipei, Taiwan; 3 Department of Pharmacy, Taipei Medical University Hospital, Taipei, Taiwan; 4 Department of Clinical Pharmacy, School of Pharmacy, Taipei Medical University, Taipei, Taiwan; 5 Division of Nephrology, Department of Internal Medicine, Shuang-Ho Hospital, Taipei Medical University, New Taipei City, Taiwan; 6 Department of Internal Medicine, School of Medicine, Taipei Medical University, Taipei, Taiwan; 7 Clinical Research Center, Taipei Medical University Hospital, Taipei, Taiwan; 8 Department of Pharmacy, Taipei Medical University-Shuang Ho Hospital, Taipei, Taiwan; 9 The Ph.D. Program for Translational Medicine, College of Medical Science and Technology, Taipei Medical University and Academia Sinica, Taipei, Taiwan; 10 HudsonAlpha Institute for Biotechnology, Huntsville, AL USA; 11 Master Program for Clinical Pharmacogenomics and Pharmacoproteomics, School of Pharmacy, Taipei Medical University, Taipei, Taiwan

**Keywords:** Cardiovascular, Endothelin, Renal failure

## Abstract

**Background:**

Cardiovascular (CV) complications are the main cause of death in end-stage renal disease (ESRD) patients. The high CV risks are attributable to the additive effects of multiple factors. Endothelin (EDN) is a potent vasoconstrictor and plays a role in regulating vascular homeostasis. However, whether variants of the *EDN* gene are associated with risks of CV events is not known. We conducted a study to investigate associations of variants of the *EDN* gene with CV events in ESRD patients.

**Methods:**

A cohort of 190 ESRD patients was recruited, and 19 tagged single-nucleotide polymorphisms within the *EDN* gene family were selected for genotyping through a TaqMan assay. Data on clinical characteristics and hospitalizations for CV events were collected. Associations of genetic variants of the *EDN* gene with CV events were analyzed.

**Results:**

In this cohort, 62% (*n* = 118) of patients were hospitalized for a CV event. The *EDN1* rs4714384 (CC/TC vs. TT) polymorphism was associated with an increased risk of a CV event after multiple testing (*p* < 0.001). Further functional exploration showed that it was a quantitative trait locus which may significantly alter gene expression in the tibial artery.

**Conclusions:**

*EDN1* rs4714384 is very likely an important biomarker of CV events in ESRD patients.

**Electronic supplementary material:**

The online version of this article (10.1186/s12882-017-0707-2) contains supplementary material, which is available to authorized users.

## Background

End-stage renal disease (ESRD) patients have a high risk of mortality, and 50% of these deaths are from cardiovascular (CV) complications [1]. Sudden cardiac death is observed in half of those cases, which is much more than coronary artery disease (CAD) [2]. Left ventricular hypertrophy (LVH) is considered to be one of the culprit pathophysiological expressions. It may contribute to the excess risk of sudden cardiac death and indicates poor survival in ESRD patients [3]. Besides LVH, vascular disorders, including atherosclerosis and arteriosclerosis, also account for the high risk of CV complications [4]. The exact mechanisms responsible for the excess CV risk in ESRD patients are not well understood.

Traditional and non-traditional risks factors, such as hypertension, diabetes, dyslipidemia, anemia, uremia, chronic inflammation, oxidative stress, calcium-phosphate vascular calcification, and autonomic dysfunction, contribute to a proportion of the excess risk [5, 6]. However, combining these risk factors does not fully explain the excess risk in these patients [5, 7]. Imbalances in some humoral factors and regulatory systems may also account for the excess CV risk. Activation of the renin-angiotensin-aldosterone system, and imbalanced endothelin (EDN) and nitric oxide levels were reported [8]. Demuth et al. [9] reported that an increased plasma endothelin level was associated with LVH and arterial intima-media thickening, suggesting this humoral factor may be important in cardiovascular remodeling. Whether genetic variants of candidate genes are related to the risk of CV disease (CVD) in ESRD patients is not known.

The *EDN* peptide family includes three isoforms (ET1, ET2, and ET3), which are coded by different genes, located on chromosomes 6p24.1 (*EDN1*), 1p34 (*EDN2*), and 20q13.2 ~ 13.3 (*EDN3*) [10]. Of these isoforms, ET1 is predominant. ET1 was reported to be strongly correlated with CVD, since it has a predominant vasoconstriction effect and hypertensive effect [10]. It has myocardial hypertrophic effects, [11] and its expression in endothelial tissues may be associated with instability of atherosclerotic plaque [12]. In addition, Minami et al. [13] reported that the plasma ET1 level was correlated with asymptomatic lacunar infarct and carotid plaques. ET2 was reported to be necessary for normal ovulation [14] and is associated with breast tumor invasion [15]. ET3 was identified as being abundant in the intestines and pituitary/brain tissues, which indicates it may have functions in nervous and endocrine systems [16]. Associations of genetic variants of the *EDN* genes with CV complications in ESRD patients are not well known. Thus, we investigated genetic variants of the *EDN* genes and CV events. We hypothesized that common variants of the *EDN* genes are associated with increased risks of hospitalizations for CV events in ESRD patients.

## Methods

### Study subjects

This study recruited adult patients older than 18 years, who had received chronic dialysis for at least 3 months at Taipei Medical University Hospital. 90% (*n* = 171) of patients received hemodialysis and the remaining received peritoneal dialysis. Demographics and clinical data of all patients were collected, including dialysis vintage, smoking, the erythropoietin resistance index (ERI), hemoglobin, albumin, iron profiles, adequacy of dialysis (Kt/V), pre-existing CV comorbidities, and cause of ESRD. ERI was calculated by the average weekly erythropoietin dose per kg body weight per average hemoglobin, which indicated a patient’s response to erythropoietin. After enrollment, patients were followed up until the development of hospitalization for a CV event. The length of time from study enrollment to the development of CV event was collected. We defined hospitalized “CV events” as including CAD, congestive heart failure (CHF), arrhythmia, aortic aneurysm, stroke, and peripheral arterial occlusive disease (PAOD). These outcomes were prespecified in our protocol. CAD was defined as patients who received coronary angiography and ≥75% stenosis of a major coronary artery was noted. Congestive heart failure was documented according to the clinical diagnostic criteria by cardiologist, either by an episode of pulmonary edema, systolic dysfunction by cardiac sonography or cardiomegaly. Arrhythmia was documented as newly onset of irregular heart beat by electrocardiogram. Aortic aneurysm was confirmed by aortic imaging via radiographic studies. Stroke was defined as focal neurologic symptoms with image evidences. Peripheral arterial occlusive disease was documented by symptoms of ischemic muscle pain and radiographic studies. This study was approved by the Institutional Review Board of Taipei Medical University (no. 201309026). Written informed consent was obtained from all patients.

### Genotyping

These tagged SNPs were determined to have a minimum allele frequency of >1% in a Beijing Han Chinese population (https://www.ncbi.nlm.nih.gov/variation/news/NCBI_retiring_HapMap/). Genotyping was done using the TaqMan Allelic Discrimination Assay (Applied Biosystems, Foster City, CA). Polymerase chain reaction (PCR) was carried out with an ABI StepOnePlus Thermal Cycler (Applied Biosystems). The fluorescence from different probes was detected and analyzed via the System SDS software version 2.2.2 (Applied Biosystems).

### Statistical analysis

R 3.2.0 was used for the statistical analyses. The Chi-squared test and Student’s *t*-test were used for comparing demographic characteristics between groups as indicated. A multivariable logistic regression model was performed to control for possible confounding factors, including age, gender, smoking, diabetes, hypertension, pre-existing CV comorbidities, hemoglobin, albumin, ferritin, and the ERI. We analyzed the magnitude of the association between the different genotypes and hospitalization for a CV event through a likelihood ratio test. Odds ratios (OR) with 95% confidence intervals were determined. Multiple testing correction was carried out using the false discovery rate (FDR), and q-values of <0.05 were determined to indicate statistical significance.

### SNP functional annotation

In order to evaluate the relationship between the SNPs and gene expression profiles, we queried the GTEx Portal (https://www.gtexportal.org/home/), which contain expression quantitative trait loci (eQTLs) across multiple tissues. The SNP function prediction web site (https://snpinfo.niehs.nih.gov/snpinfo/snpfunc.html), which provides a variety of possible downstream influence of variants, was also applied to identify potential impact of candidate SNPs.

## Results

**Fig. 1:**
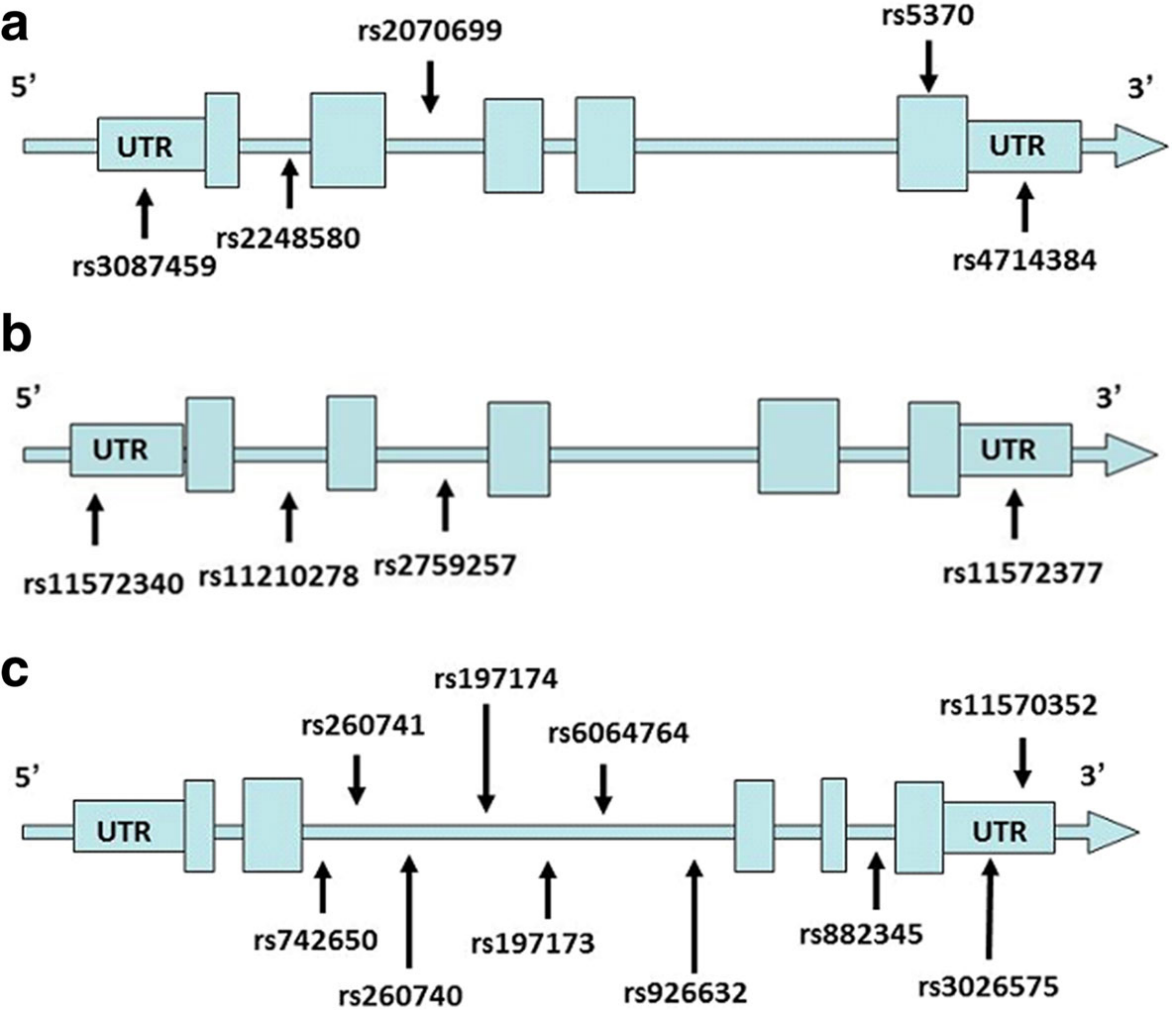
**a**. Graphic view of the genotyped human EDN-1 gene. **b**. Graphic view of the genotyped human EDN-2 gene. **c**. Graphic view of the genotyped human EDN-3 gene

In total, 19 tSNPs of the *EDN* family (*EDN1*: rs5370, rs2070699, rs2248580, rs4714384, and rs3087459; *EDN2*: rs2759257, rs11210278, rs11572340, and rs11572377; and *EDN3*: rs742650, rs260740, rs260741, rs6064764, rs197173, rs197174, rs882345, rs926632, rs3026575, and rs11570352) were genotyped **(**Fig. 1a-c **).** The minor allele frequencies of these tSNPs were close to those of the reference population from the Taiwan Biobank database and HapMap project (Table 1).

**Table 1:** Minor allele frequencies of selected tagged single-nucleotide polymorphisms (SNPs) of the endothelin (EDN) gene family

Gene	Position	SNP	Location	Ref	Alt	AFR fre	AMR fre	ASN fre	EUR fre	TWB fre	Our fre
*EDN1*	Ch6:12,289,406	rs3087459	5’UTR	A	C	0.19	0.14	0.20	0.20	0.23	0.17
	Ch6:12,291,749	rs2248580	Intron	C	A	0.08	0.56	0.56	0.47	0.41	0.35
	Ch6:12,292,539	rs2070699	Intron	G	T	0.05	0.46	0.56	0.47	0.49	0.43
	Ch6:12,296,022	rs5370	Missense	G	T	0.14	0.17	0.28	0.21	0.31	0.23
	Ch6:12,297,620	rs4714384	3’UTR	T	C	0.71	0.38	0.62	0.37	0.38	0.34
*EDN2*	Ch1:41,485,234	rs11572340	5’UTR	C	A	0.04	0.16	0.07	0.21	0.06	0.02
	Ch1:41,484,301	rs11210278	Intron	C	T	0.01	0.11	0.33	0.19	0.30	0.29
	Ch1:41,483,957	rs2759257	Intron	A	C	0.75	0.89	0.91	0.86	0.88	0.93
	Ch1:41,478,124	rs11572377	3’UTR	C	G	0.01	0.01	0.08	0.02	0.09	0.05
*EDN3*	Ch20:59,303,616	rs742650	Intron	C	T	0.00	0.03	0.15	0.06	0.14	0.11
	Ch20:59,301,100	rs260741	Intron	G	A	0.06	0.31	0.24	0.21	0.25	0.27
	Ch20:59,305,927	rs260740	Intron	T	G	0.28	0.25	0.22	0.28	0.22	0.15
	Ch20:59,303,025	rs197174	Intron	T	C	0.63	0.30	0.16	0.26	0.16	0.12
	Ch20:59,303,536	rs197173	Intron	T	G	0.96	0.67	0.72	0.85	0.74	0.74
	Ch20:59,309,196	rs6064764	Intron	T	C	0.03	0.20	0.07	0.32	0.09	0.10
	Ch20:59,309,707	rs926632	Intron	C	T	0.42	0.70	0.85	0.70	0.87	0.89
	Ch20:59,319,323	rs882345	Intron	A	G	0.10	0.16	0.15	0.19	0.13	0.09
	Ch20:59,324,630	rs3026575	3’UTR	G	A	0.00	0.00	0.05	0.00	0.04	0.14
	Ch20:59,324,605	rs11570352	3’UTR	C	T	0.00	0.00	0.05	0.00	0.06	0.06

*Fre* Alt frequency, *UTR* untranslated region

In total, 190 patients were recruited for this study. The length of follow-up for the entire cohort was 22.9 +/− 10.6 months. The mean age was 64 years, and 54% of them were male. Pre-existing CV events were found in 43 patients with more than 83% due to CHF (*n* = 16, 37%) and CAD (*n* = 20, 47%). After enrollment, there were 118 patients (62.1%) who experienced hospitalization for a CV event with a mean duration of 17.2 ± 8.8 months. Of these CV events, CAD and CHF remained the most commonly observed (Additional file 1: Table S1). We divided patients into study and control groups according to the development of CV events. Patients in the study group included more males, were older, and had more diabetes. Lower albumin, serum iPTH, and Kt/V levels were observed in study group patients (Table 2).

**Table 2:** Baseline characteristics of study patients according to cardiovascular disease (CVD)

	CVD (*n* = 118)	No CVD (*n* = 72)	*p* value
Gender, male, *n* (%)	74 (62.7%)	29 (40.3%)	**0.003**
Age (years)	67 ± 13	60 ± 12	**<0.001**
Dialysis vintage (years)	4.3 ± 3.8	5.5 ± 6.7	0.111
Current smoking (%)	20 (16.9%)	5 (6.9%)	0.050
Diabetes, *n* (%)	73 (61.9%)	17 (23.6%)	**<0.001**
ERI (unit/week/kg/Hb)	8.5 ± 6.7	8.0 ± 4.1	0.600
Hemoglobin (g/dl)	10.7 ± 1.2	10.7 ± 1.1	0.980
Albumin (g/dl)	3.9 ± 0.4	4.1 ± 0.4	**0.005**
Ferritin (mg/dl)	418 ± 401	458 ± 736	0.635
Iron (mg/dl)	65 ± 24	68 ± 33	0.571
TIBC (mg/dl)	236 ± 47	242 ± 54	0.441
Serum i-PTH (pg/mL)	277 ± 363	422 ± 354	**0.012**
Kt/V	1.44 ± 0.26	1.60 ± 0.31	**0.001**
Cause of ESRD, n (%)			**<0.001**
Hypertension	16 (13.5%)	18 (25.0%)	
Diabetes	67 (56.8%)	17 (23.6%)	
GN	14 (11.9%)	22 (30.6%)	
CHF	8 (6.8%)	0 (0%)	
Others	13 (11.0%)	15 (20.8%)	

*CHF* congestive heart failure, *ERI* erythropoietin resistance index, *ESRD* end-stage renal disease, *GN* glomerulonephritis, *iPTH* parathyroid hormone, *Kt/V* dialysis adequacy, *TIBC* total iron-binding capacity. *p* values of <0.05 are shown in bold

After multivariable adjustment, we found that patients carrying *EDN1* rs2248580 (AA/CA vs. CC), rs2070699 (GT/TT vs. GG), rs4714384 (CC/TC vs. TT) (Table 3), *EDN2* rs11210278 (TC/CC vs. TT) (Table 4) were associated with an increased risk of being hospitalized for a CV event in the recessive models. All of the *EDN1* variants remained statistically significant after multiple testing correction (q values of 0.013, 0.003, and <0.001). However, none of *EDN3* SNPs showed significant association with the susceptibility of developing cardiovascular disease (Table 5). In addition, we conducted functional annotation for these SNPs via several publicly available databases. The results showed that *EDN1* rs4714384 is an eQTL in the tibial artery tissue (*p* = 6.3*10^−9^) (Table 6). We further analyzed the association of *EDN1* rs4714384 with each CV comorbidity, which showed non-significant findings **(**Additional file 1: Table S2**)**.

**Table 3:** Association analysis of genetic polymorphisms of the EDN1 gene and cardiovascular disease susceptibility in end-stage renal disease patients

		Cardiovascular disease susceptibility			Recessive	
rs number	Genotype	Cases (%)	Control (%)	OR (95% CI)	*p* value	q-value
rs3087459	CC	3 (3%)	3 (5.8%)	0.06 (0.01–0.69)	**0.019**	0.086
	CA	26 (25.7%)	13 (25%)	1		
	AA	72 (71.3%)	36 (69.2%)	1		
rs2248580	CC	8 (7.8%)	12 (21.8%)	0.10 (0.02–0.47)	**0.001**	**0.013**
	CA	49 (48.0%)	22 (40.0%)	1		
	AA	45 (44.1%)	21 (38.2%)	1		
rs2070699	GG	11 (11.7%)	16 (29.6%)	0.09 (0.02–0.38)	**<.001**	**0.003**
	GT	50 (53.2%)	22 (40.7%)	1		
	TT	33 (35.1%)	16 (29.6%)	1		
rs5370	TT	5 (2.5%)	4 (10.0%)	0.30 (0.04–2.08)	0.216	0.388
	TG	32 (40.0%)	20 (32.2%)	1		
	GG	61 (57.5%)	28 (57.8%)	1		
rs4714384	TT	7 (7.2%)	14 (26.9%)	0.05 (0.01–0.23)	**<.001**	**<.001**
	TC	42 (43.3%)	17 (32.7%)	1		
	CC	48 (49.5%)	21 (40.4%)	1		

*p* values were adjusted for age, sex, smoking, diabetes, hypertension, pre-existing cardiovascular events, hemoglobin, albumin, ferritin, and the erythropoietin resistance index. *p* and q-values of <0.05 are shown in bold. q-values of <0.05 were considered statistically significant after correction for multiple testing. OR, odds ratio; CI, confidence interval

**Table 4:** Association analysis of genetic polymorphisms of the EDN2 gene and cardiovascular disease susceptibility in end-stage renal disease patients

		Cardiovascular disease susceptibility			Recessive	
rs number	Genotype	Cases (%)	Control (%)	OR (95% CI)	*p* value	q-value
rs11572340	AA	1 (0.9%)	0 (0%)	0.74 (0.14 ~ 3.85)	0.708	0.796
	AC	5 (4.3%)	2 (2.9%)	1		
	CC	109 (94.8%)	66 (97.1%)	1		
rs11210278	TT	8 (9.9%)	7 (18.9%)	0.17 (0.03 ~ 0.88)	**0.035**	0.126
	TC	30 (37.0%)	9 (24.3%)	1		
	CC	43 (53.1%)	21 (56.8%)	1		
rs2759257	AA	0 (0%)	0 (0%)	-	NA	NA
	AC	17 (15.3%)	6 (9.4%)	1		
	CC	94 (84.7%)	58 (90.6%)	1		
rs11572377	GG	2 (1.7%)	1 (1.4%)	0.88 (0.07 ~ 11.11)	0.920	0.953
	GC	10 (8.6%)	2 (2.8%)	1		
	CC	104 (89.7%)	69 (95.8%)	1		

*p* values were adjusted for age, sex, smoking, diabetes, hypertension, pre-existing cardiovascular events, hemoglobin, albumin, ferritin, and the erythropoietin resistance index. *p* and q-values of <0.05 are shown in bold. q-values of <0.05 were considered statistically significant after correction for multiple testing. OR, odds ratio; CI, confidence interval

**Table 5:** Association analysis of genetic polymorphisms of the EDN3 gene and cardiovascular disease susceptibility in end-stage renal disease patients

		Cardiovascular disease susceptibility			Recessive	
rs number	Genotype	Cases (%)	Control (%)	OR (95% CI)	*p* value	q-value
rs742650	TT	0 (0%)	2 (3.9%)	-	0.087	0.197
	CT	20 (21.5%)	7 (13.7%)	1		
	CC	73 (78.5%)	42 (82.4%)	1		
rs260741	AA	8 (8.4%)	4 (9.1%)	0.45 (0.08 ~ 2.44)	0.356	0.583
	AG	31 (32.6%)	19 (43.2%)	1		
	GG	56 (58.9%)	21 (47.7%)	1		
rs260740	GG	1 (1.0%)	0 (0%)	-	0.708	0.796
	GT	27 (26.0%)	16 (32.0%)	1		
	TT	76 (73.1%)	34 (68.0%)	1		
rs197174	GG	1 (1.0%)	4 (7.7%)	0.12 (0.01 ~ 1.49)	0.074	0.197
	GA	19 (18.4%)	9 (17.3%)	1		
	AA	83 (39.0%)	39 (75.0%)	1		
rs197173	TT	9 (9.0%)	6 (11.1%)	1.67 (0.38 ~ 7.14)	0.497	0.662
	GT	33 (33.0%)	18 (33.3%)	1		
	GG	58 (58.0%)	30 (55.6%)	1		
rs6064764	CC	2 (1.9%)	1 (1.6%)	0.03 (0.01 ~ 1.59)	0.083	0.197
	CT	24 (22.4%)	4 (6.3%)	1		
	TT	81 (75.7%)	58 (92.1%)	1		
rs926632	CC	2 (1.9%)	3 (5.2%)	2.86 (0.97 ~ 8.33)	0.514	0.662
	CT	19 (17.8%)	8 (13.8%)	1		
	TT	86 (80.4%)	47 (81.0%)	1		
rs882345	GG	2 (1.9%)	2 (3.7%)	0.92 (0.05 ~ 16.67)	0.953	0.953
	GA	14 (13.5%)	7 (13.0%)	1		
	AA	88 (84.6%)	45 (83.3%)	1		
rs3026575	AA	1 (1.0%)	0 (0%)	-	0.515	0.662
	AG	7 (6.8%)	4 (7.1%)	1		
	GG	95 (92.2%)	52 (92.9%)	1		
rs11570352	TT	3 (2.5%)	5 (7.0%)	0.20 (0.01 ~ 2.94)	0.216	0.388
	TC	4 (3.4%)	1 (1.4%)	1		
	CC	111 (94.1%)	65 (91.5%)	1		

*p* values were adjusted for age, sex, smoking, diabetes, hypertension, pre-existing cardiovascular events, hemoglobin, albumin, ferritin, and the erythropoietin resistance index. q-values of <0.05 were considered statistically significant after correction for multiple testing. OR, odds ratio; CI, confidence interval

**Table 6:** Endothelin (EDN) gene family-related expression quantitative trait loci (eQTLs)

Gene symbol	SNP Id	GENCODE ID	*p* value	Effect size	Tissue
*EDN1*	rs3087459	ENSG00000078401.6	1.5e-5	0.25	Cells - Transformed fibroblasts
*RN7SKP293*	rs4714384	ENSG00000223321.1	6.3e-9	−0.37	Artery - Tibial
*EDN2*	rs11210278	ENSG00000127129.5	3.1e-6	0.55	Heart - Left Ventricle

## Discussion

In this study, we systemically performed genotyping of the *EDN* gene family, and three variants in the *EDN1* gene [rs2248580 (AA/CA vs. CC), rs2070699 (GT/TT vs., GG), and rs4714384 (CC/TC vs. TT)] were associated with an increased risk of a CV event. These tSNPs were not located in the exon region, and therefore were not correlated with protein-coding functions. These genetic variants may alter disease phenotypes through other pathways, such as non-coding RNA, transcriptional regulation, or alterations in splicing [17]. Although there is no eQTL evidence between rs4714384 and *EDN1* being observed in the current database due to limited available tissue specific profile, we found that rs4714384 has impact on *RN7SKP293* expression in the tibial artery. The *RN7SKP293* is a pseudogene, belonging to the 7SK RNA class. 7SK RNA is found in a small nuclear ribonucleoprotein (snRNP) complex, which regulates the activity of positive transcription elongation factor b (P-TEFb) [18]. P-TEFb is a kind of cyclin-dependent kinase (Cdk) which controls the elongation phase of transcription by RNA polymerase II [19]. Cdk is a cell-cycle check point regulator, and one study showed Cdk9 may have transcriptional roles in cardiac hypertrophy and mitochondrial dysfunction [20]. Another study showed overexpression of Cdk2 may promote smaller, less differentiated cardiomyocytes which have increased response to pressure overload [21]. Cdk dysregulation may be related to LVH, which may result in future CHF. Endothelial cells are the main origin of ET-1 production. Endothelial dysfunction is an important pathophysiology in ESRD patients, and is strongly associated with a risk of atherosclerosis and consequent CV events [22]. Its manifestations represent a systemic pathogenic condition, which implies an inflammatory state, prothrombotic state, and impaired vasomotor and cellular proliferation in the vascular wall [23]. Imbalances of humoral factors, including nitric oxide, oxidative stress, chemokines, angiotensin II, and EDN-1, on vascular homeostasis may contribute to this condition [22]. In addition, Ganz et al. [24] reported that the peripheral artery endothelial function has rather better prognostic predictions of CV events than coronary artery endothelial function. Our findings suggest that genetic variants of *EDN1* may alter the balance of the homeostasis of peripheral vascular regulation, and affect the susceptibility to CV comorbidities.

We further investigated *EDN* gene expressions among different tissues through GTEx. However, low expression levels of both *EDN2* and *EDN3* were noted in the heart and major vessel tissues [25]. By querying the SNP function prediction web site, we found that rs11210278 (caTAAT**C**gag) is a potential binding site for GATA6, which is a transcription factor involved in hypertrophic cardiomyopathy (Additional file 1: Table S3). This finding may be correlated with our finding, since the C allele of the rs11210278 is a predominant binding site for GATA6; therefore, patients carrying this allele may confer an increased binding affinity of the GATA6 transcription factor and consequently increased risk of hypertrophic cardiomyopathy. Cardiomyopathy is an important pathophysiology of CV events in ESRD patients [5]. Although this allele was not statistically significant after multiple testing correction, further enlarging sample sizes and functional validation studies are warranted for confirmation.

Vargas-Alarcon et al. [26] reported that an *EDN1* rs3087459 polymorphism (AA allele) was associated with an increased risk of developing acute coronary syndrome. However, two other studies did not find such an association of *EDN1* rs3087459 with the risk of myocardial infarction or ventricular hypertrophy [27, 28]. In our study, we also did not find a statistically significant association of higher risk of CV events with the SNP rs3087459. Rankinen et al. [29] reported that an *EDN1* (Glu106Glu) polymorphism had a risk of HTN in a Caucasian population. Another study showed that genetic polymorphisms of the *EDN1* rs5370 T allele and rs2070699 G allele were associated with an increased risk of ischemic stroke, [30] which is contrary to our findings. We found that rs3087459 may alter the gene expression in transformed fibroblasts according to the results of eQTL database; however, the association of fibroblasts with CV events remained elusive. Comparing the above studies to ours, different outcome measurements were noted. The small sample size may also limit our observations.

Previous studies showed dialysis vintage is a risk factor for coronary artery calcification, which may reflect the major risk of CV events [31, 32]. Therefore, the increased risk of CV events occurred with a longer duration of dialysis, which may account for the increased events during our observation period. According to the USRDS 2012 annual data report, [33] the incidence of hospitalization for CV morbidity is 4.5–5 times per 100-patient-months. Our cohort showed a relative fewer hospitalization of CV events, as compared to US database. We have a better 5-year overall survival in dialysis patients. Compared to other countries, the CV-related morbidity and mortality are much lower in Taiwan [34]. It may reflect the different epidemiology of CV events among populations. In our study, being male, being elderly, having hypoalbuminemia, and having underlying diabetes mellitus were risk factors for CV events. Being male and elderly are well known risk factors for CVDs [35, 36]. Malnutrition-inflammation complex syndrome is frequently observed in dialysis patients and is associated with an increased risk of CVD. Protein energy wasting and low albumin levels are poor outcome indicators [37]. Diabetes patients exhibit increased insulin resistance and an inflammatory status [38]. A previous study by Chang et al. [39] reported that diabetes and ESRD synergistically contribute to an increased risk of CV events.

Lower PTH and Kt/V level were associated with increased risk of CV events. Some studies showed hyperparathyroidism was associated with increased risk of CV mortality and all-cause mortality in dialysis patients [40–42]. However, a meta-analysis showed no significant association between PTH level and non-fatal cardiovascular events or CV mortality [43]. The PTH level is pulsatile in character and highly sensitive to change in ionized calcium and calcitriol levels [44]. There may be selection bias in our small sample size cohort. Therefore the association between low PTH and risk of CV events may need further investigation. In renal failure patients, there are a lot of uremic toxins leading to inflammation and subsequently CV events. Better Kt/V is associated with less uremic toxins retention, which may be related to lower risk of CV events. Đurić PS et al. [45] reported longer dialysis duration was associated with lower CV comorbidities. The FHN (Frequent Hemodialysis Network) trial group [46] also found better composite outcomes of death or change in left ventricular mass in frequent hemodialysis group as compared to conventional hemodialysis. In this view, the patients who received better dialysis clearance may have lower risk of CV events.

As we know, there were several identified risk factors contributing to cardiovascular comorbidities in renal failure patients. However, the prevalence and severity of CV events in ESRD patients is disproportionate to the identified risk profiles [47]. In the current study, we found potential genetic risk alleles and this may further improve the prediction model for CV comorbidities in these patients.

Several limitations of this study should be noted. First, the sample size was not large, and thus the statistical power may be limited. Second, we did not check the plasma ET-1 level to prove associations of genetic variants with its expression, as it was reported to be associated with several phenotypes in previous studies [13]. Third, we did not find the association between the SNPs of EDN and pre-existing CV events. It may be due to different composition of pre-existing CV events and different dialysis stage of these patients.

## Conclusions

We found three genetic variants of the *EDN1* gene to be associated with increased risk of hospitalization for a CV event, and rs4714384 was responsible as an eQTL in the peripheral artery. It may influence the *EDN1* gene expression and alter vascular homeostasis in the peripheral artery. A further validation study is required to confirm the roles of these polymorphisms in the risk of CV events.

## Additional file

Additional file 1: Table S1. Demographic characteristic of the study patients. **Table S2.** Association of EDN1_rs4714384 and cardiovascular disease in ESRD patients. **Table S3.** Potential function prediction of SNP in transcription factor binding site in EDN gene family. (DOCX 21 kb)

## Abbreviations

CAD: Coronary artery disease
Cdk: Cyclin-dependent kinase
CHF: Congestive heart failure
CV: Cardiovascular
EDN: Endothelin
eQTL: expression quantitative trait locus
ERI: Erythropoietin resistance index
ESRD: End stage renal disease
ET1: Endothelin 1
FDR: False discovery rate
GTEx: Genotype-Tissue Expression
Kt/V: Adequacy of dialysis
LVH: Left ventricular hypertrophy
OR: Odds ratios
PAOD: Peripheral arterial occlusive disease
PCR: Polymerase chain reaction
P-TEFb: Positive transcription elongation factor b
snRNP: small nuclear ribonucleoprotein
