# Supplementary material for: Associations of genetic variants of endothelin with cardiovascular complications in patients with renal failure
Source: BMC Nephrol. 2017 Sep 7;18:291. doi: 10.1186/s12882-017-0707-2 (PMC5590196; doi:10.1186/s12882-017-0707-2)
Supplement: Additional file 1: Table S1. — Demographic characteristic of the study patients. Table S2. Association of EDN1_rs4714384 and cardiovascular disease in ESRD patients. Table S3. Potential function prediction of SNP in transcription factor binding site in EDN gene family. (DOCX 21 kb) [file 12882_2017_707_MOESM1_ESM.docx]

| Table S1. Demographic characteristic of the study patients | |
| --- | --- |
|  | Dialysis patients |
| Number of subjects | 190 |
| Gender, male, n (%) | 103 (54%) |
| Age (years) | 64 ± 13 |
| Age range | 24-100 |
| ERI (unit/week/kg/Hb) | 8.3 ± 5.8 |
| Hospitalized CV events, n (%) | 118 (62%) |
| Coronary artery disease, n (%) | 82 (43%) |
| Congestive heart failure, n (%) | 71 (37%) |
| Stroke, n (%) | 15 (8%) |
| Arrhythemia, n (%) | 22 (12%) |
| PAOD, n (%) | 17 (9%) |
| Aortic aneurysm, n (%) | 2 (1%) |
| CV: cardiovascular; ERI: erythropoietin resistance index; PAOD: peripheral arterial occlusive disease | |

| Table S2. Association of EDN1_rs4714384 and cardiovascular disease in ESRD patients | | | | | | | |
| --- | --- | --- | --- | --- | --- | --- | --- |
| EDN1_rs4714384 | **Genotype** | **Case** |  | **Control** |  | **Recessive** | **Recessive** |
|  |  | **n** | **(%)** | **n** | **%** | **P Value** | **q value** |
| Coronary artery disease | TT  CT  CC | 5  27  30 | 8.1  43.5  48.4 | 13  27  36 | 17.1  35.5  47.4 | 0.066 | 0.586 |
| Congestive heart failure | TT  CT  CC | 5  20  28 | 9.4  37.7  52.8 | 13  34  38 | 15.3  40.0  44.7 | 0.117 | 0.586 |
| Stroke | TT  CT  CC | 2  4  5 | 18.2  36.4  45.5 | 16  50  61 | 12.6  39.4  48.0 | 0.931 | 1.000 |
| Arrhythemia | TT  CT  CC | 1  9  9 | 5.3  47.4  47.4 | 17  45  57 | 14.3  37.8  47.9 | 0.058 | 0.586 |
| PAOD | TT  CT  CC | 2  6  6 | 14.3  42.9  42.9 | 16  48  60 | 12.9  38.7  48.4 | 0.575 | 0.974 |
| Aortic aneurysm | TT  CT  CC | 0  0  1 | 0  0  100 | 18  54  65 | 13.1  39.4  47.4 | 1.000 | 1.000 |

ESRD: end stage renal disease; PAOD: peripheral arterial occlusive disease

| Table S3. Potential function prediction of SNP in transcription factor binding site in EDN gene family | | | | | |
| --- | --- | --- | --- | --- | --- |
| Gene | **rs number** | **Position** | **Forward sequence** | **TFBS** | **Function** |
| EDN1 | rs3087459 | 6 | agGCAACat | RFX | Essential expression of MHC class II genes |
| EDN2 | rs11210278 | 7 | caTAAT**C**gag | GATA6 | Involved in hypertrophic cardiomyopathy |
|  | rs11210278 | 9 | tgcataaT**C**GAGgga | CDP | Terminal differentiation, cell cycle progression |
|  | rs11210278 | 9 | tgcaTAAT**C**gagg | CRX | Differentiation of photoreceptor cells |
|  | rs11210278 | 7 | cataaT**T**GAGgg | PBX1 | Regulation of osteogenesis |
| TFBS: transcript factor binding site | | | | | |
